# Changed processing of visual sexual stimuli under GnRH-therapy – a single case study in pedophilia using eye tracking and fMRI

**DOI:** 10.1186/1471-244X-14-142

**Published:** 2014-05-17

**Authors:** Kirsten Jordan, Peter Fromberger, Helge Laubinger, Peter Dechent, Jürgen L Müller

**Affiliations:** 1 Department of Forensic Psychiatry and Psychotherapy, Georg-August-University Göttingen, Rosdorfer Weg 70, Göttingen 37081, Germany; 2 Asklepios Forensic Psychiatric Hospital of Göttingen, Rosdorfer Weg 70, Göttingen 37081, Germany; 3 Department of Cognitive Neurology, Georg-August-University Göttingen, Robert-Koch-St. 40, Göttingen 37075, Germany

**Keywords:** Pedophilia, Eye movements, Functional magnetic resonance imaging (fMRI), Subliminal, Antiandrogen therapy (ADT)

## Abstract

**Background:**

Antiandrogen therapy (ADT) has been used for 30 years to treat pedophilic patients. The aim of the treatment is a reduction in sexual drive and, in consequence, a reduced risk of recidivism. Yet the therapeutic success of antiandrogens is uncertain especially regarding recidivism. Meta-analyses and reviews report only moderate and often mutually inconsistent effects.

**Case presentation:**

Based on the case of a 47 year old exclusively pedophilic forensic inpatient, we examined the effectiveness of a new eye tracking method and a new functional magnetic resonance imaging (fMRI)-design in regard to the evaluation of ADT in pedophiles. We analyzed the potential of these methods in exploring the impact of ADT on automatic and controlled attentional processes in pedophiles. Eye tracking and fMRI measures were conducted before the initial ADT as well as four months after the onset of ADT. The patient simultaneously viewed an image of a child and an image of an adult while eye movements were measured. During the fMRI-measure the same stimuli were presented subliminally.

Eye movements demonstrated that controlled attentional processes change under ADT, whereas automatic processes remained mostly unchanged. We assume that these results reflect either the increased ability of the patient to control his eye movements while viewing prepubertal stimuli or his better ability to manipulate his answer in a socially desirable manner. Unchanged automatic attentional processes could reflect the stable pedophilic preference of the patient. Using fMRI, the subliminal presentation of sexually relevant stimuli led to changed activation patterns under the influence of ADT in occipital and parietal brain regions, the hippocampus, and also in the orbitofrontal cortex. We suggest that even at an unconscious level ADT can lead to changed processing of sexually relevant stimuli, reflecting changes of cognitive and perceptive automatic processes.

**Conclusion:**

We are convinced that our experimental designs using eye tracking and fMRI could prospectively add additional and valuable information in the evaluation of ADT in paraphilic patients and sex offenders. But with respect to the limited significance of this single case study, these first results are preliminary and further studies have to be conducted with healthy subjects and patients.

## Background

### Antiandrogen treatment in pedophilic patients

Following the American Psychiatric Association (APA), pedophilia (a subtype of paraphilia) is defined as a recurrent sexual interest in prepubescent children, characterized by persistent thoughts, fantasies, urges, sexual arousal or behavior [1]. Current therapeutic approaches include psychotherapy as well as pharmacological treatment, among which the androgen-lowering therapy [2]. Antiandrogen drugs, such as cyproterone acetate, medroxyprogesterone acetate or the more recently introduced gonadotropin-releasing hormone agonists (GnRH agonist), are utilized to drastically decrease the testosterone concentration in patients. ADT is also known as "chemical castration" due to the fact that serum testosterone concentration can drop rapidly and even reach castration levels after several weeks. The aim of the treatment is a reduction in sexual drive and, in consequence, a reduced risk of recidivism in paraphilic patients and sexual offenders.

It has been demonstrated that testosterone modulates not only autonomic sexual functions such as erection and ejaculation, but also motivational, cognitive and emotional aspects of sexual functions, such as sexual interest, thoughts and fantasies. Accordingly, a decrease of sexual motivation, intensity and frequency of deviant sexual fantasies as well as a decrease of the masturbation rate has been described in paraphilic patients and sex offenders treated with antiandrogens [3,4]. Despite a large number of studies, the efficiency of ADT aiming at a reduction of the sex drive and a reduced risk of recidivism is still under debate. Meta-analyses and reviews report only moderate and often mutually inconsistent effects [4-9]. Only a few placebo-controlled double blind studies have been performed with inconsistent results concerning treatment effects [10-20]. Furthermore, outcome measures differ between these studies and do not seem to be ideally suited to their purpose. Most studies measured the concentration of testosterone, luteinizing hormone, follicle-stimulating hormone and other physiological parameters, or used self-reports about sexual activity and interest or recidivism rate [4]. But Schober, Kuhn, Kovacs, Earle, Byrne and Fries [19] have pointed out that self-reporting by patients does not seem to be reliable.

### The assessment of pedophilic preference

Self-reports about sexual interest are the diagnostic standard in Western Europe, irrespective of its insufficient validity and reliability due to the tendency of participants to answer in a socially desirable manner [21]. But the strongest single predictor for sexual offence recidivism is the existence of deviant sexual preferences [22]. Penis plethysmography (PPG) is the gold standard in assessing pedophilic interest in North America and has demonstrated good classification accuracy [23-25]. Nevertheless, the application of PPG is limited by certain factors and still controversially discussed [26]. Recent methods try to assess pedophilic sexual interest applying cognitive approaches [27], e.g. the implicit association test (IAT) [28], the viewing time (VT) [29,30], the choice-reaction-time task (CRT); [31], the rapid serial visual presentation test (RSVP); [32] or an adaption of the emotional stroop task for sexual offenders [33].

Recently, Fromberger et al. [34] introduced another assessment method by measuring automatic and controlled attentional processes while viewing pedophilic stimuli. Based on the assumption of Spiering et al. [35] that sexually relevant features of a stimulus are preattentively selected and automatically trigger focal attention to these sexual aspects, the authors illustrated that the eye tracking method provides the possibility to measure the assumed bias in initial orienting and the maintenance of attention to pedophilic stimuli in pedophiles [36,37].

### Functional imaging for the assessment of sexual interest in healthy subjects and pedophilic patients

For healthy subjects it has been shown that the supraliminal presentation of visual sexual stimuli evokes hemodynamic responses in a complex neural network. Based on the results of more than 70 functional imaging studies a descriptive four-component model of sexual arousal was proposed implicating four excitatory components: a cognitive, an emotional, a motivational and an autonomic/endocrinological component [38,39]. The model and the associated brain regions are presented in detail in Additional file 1: Figure S1 (see 1. Introduction: The four-component model). Although, not all studies report brain activation in the above mentioned regions consistently, the four-component model reveals the complexity of the neural network linked with sexual arousal.

Several studies examined brain activation in pedophilic subjects while presenting sexually relevant visual stimuli (for overview: [4,40]). Most of them used a passive stimulation design, presenting visual sexual stimuli for several seconds, e.g. at a supraliminal level. Comparing the brain regions described in the four-component model with the results of the studies in pedophilic patients it can be summarized that functional changes in pedophilia seem to occur predominantly in brain regions involved in sexual functions, such as the frontal, temporal, parietal or occipital cortical regions, the limbic circuits, and subcortical areas such as the hypothalamus, the insula, or the basal ganglia [41-50]. It can be concluded that the presentation of sexually relevant stimuli, i.e. images of girls or boys in pedophilic patients evoke similar brain activation like sexually relevant stimuli in healthy non-pedophilic subjects (i.e. images of women and men). However, restrictively it has to be mentioned that these functional imaging studies applied different paradigms, compared different patient groups, and used different comparisons between experimental conditions or groups, impeding direct comparison between these studies.

### Functional imaging and antiandrogen therapy in pedophilic patients

As mentioned above, testosterone modulates not only autonomic sexual functions but also motivational, cognitive and emotional aspects of sexual functions. Considering the distribution of androgen receptors (AR) in the brain this becomes comprehensible. Amongst others AR’s have been found in the hypothalamus, the amygdala, the hippocampus and in several cortical regions in animals and partially in humans. Furthermore, a correlation between testosterone and hemodynamic responses or rCBF has been shown in frontal, temporal, parietal and occipital regions as well as in the limbic system in healthy and in hypogonadal men (for an overview see: [3]). Most brain regions were implemented in the four-component model (see Additional file 1: Figure S1, [38]). It has been assumed that testosterone modulates sexual functions through the stimulation of dopamine release in brain regions associated with sexual arousal [51,52]. Thus, if testosterone concentration declines during ADT, the activity in those brain regions should decrease in correspondence with the decrease of cognitive/attentional, emotional, motivational and autonomic reactions in response to sexual stimuli.

To our knowledge, only three single case studies are published which examined the effect of an ADT by using fMRI [53-55]. All three studies used similar experimental passive designs, presenting sexually relevant stimuli at a supraliminal level. Sexually relevant stimuli were images of girls or boys for the pedophilic patients and images of women or men for healthy controls. Schiffer et al. compared a heterosexual pedophilic patient, treated with a GnRH agonist, with a group of unmedicated heterosexual pedophiles [53]. The patient showed lower or no activation in brain regions associated with the emotional or autonomic component of sexual arousal. In the second study a homosexual pedophilic patient was compared before and five months after the onset of the GnRH agonist treatment with an age-matched heterosexual healthy man [54]. Only the pedophile showed a decrease of hemodynamic responses under GnRH therapy in the left calcarine fissure, left insula, ACC and left cerebellar vermis. The authors suggest that GnRH treatment decreased the activation in regions known to mediate the perceptual, motivational and affective responses to visual sexual stimuli [54]. Finally, Habermeyer et al. examined a homosexual pedophilic patient before and 10 months after the onset of a GnRH treatment [55]. The patient showed a decrease of hemodynamic responses under treatment in the right amygdala, the right superior frontal gyrus, the right precentral gyrus and the right superior temporal gyrus. Besides the limitations of these three case studies it can be assumed that an ADT can lead to a decreased activation in brain regions linked with sexual functions, esp. with the autonomic, the emotional and the motivational component of sexual arousal while viewing supraliminally presented pedophilic stimuli. But a differentiation between automatic and controlled attentional processes while viewing sexual stimuli is not possible based on these experimental designs. This could be accomplished by presenting pedophilic stimuli subliminally, since the subject will not consciously know the pedophilic content. Until now it is unclear if subliminally presented pedophilic stimuli elicit similar brain activation such as the above described supraliminally presented stimuli. Moreover, it is not clear if ADT has an impact on subliminally elicited brain responses while viewing sexually relevant stimuli.

### Processing of subliminally presented sexual stimuli

According to recent definitions stimuli are considered as subliminal if they are processed by the brain, but not consciously perceived [56]. Subliminal perception can be achieved by presenting stimuli usually no longer than 50 ms, followed by a masking procedure. Subliminal stimuli are also used as primers, in which primers influence the future conscious action without awareness of the prime [56]. Supporting the model of Spiering and Everaerd [35] it was shown that subliminally presented sexual primes elicit erectile responses [57] and also facilitate the identification of sexual targets in men [58]. Thus, subliminally presented sexual stimuli can trigger the automatic cognitive process inducing implicit memory processes and subsequent physiological arousal. Similarly, conscious cognitive elaboration of sexual stimuli is essential to experience subjective arousal given the result that subliminally presented primes did not elicit subjective sexual arousal [58,59]. Recently, Brooks et al. [56] published a meta-analysis of functional imaging studies investigating the processing of subliminally presented arousing stimuli (faces, physiological, lexical and audio stimuli). They found a network involving primary visual brain areas, somatosensory regions as well as implicit memory areas and conflict monitoring brain regions, representing a state which is at first independent of conscious processing.

To our knowledge only three studies examined the hemodynamic responses of subliminally presented visual sexual stimuli. None of these studies were included in the meta-analysis of Brooks, Savov, Allzén, Benedict, Fredriksson and Schiöth [56]. These studies showed that subliminally presented sexual stimuli can evoke brain activation in regions known to be activated in response to other subliminally presented arousing stimuli, e.g. in the occipital cortex, amygdala, insula and also in the cingulate cortex. Furthermore, activation in the OFC and in frontal, temporal, parahippocampal and parietal regions was reported [60-62].

Including the results of psychological priming studies, the results of the imaging studies using subliminal stimuli and the four-component model of sexual arousal, we suppose that subliminally presented visual sexual stimuli can elicit hemodynamic responses in brain regions associated with the autonomic component (insula, ACC), the emotional component (amygdala), the motivational component (ACC, parietal cortex), and supposedly the cognitive component (right lateral OFC, parietal cortex). The latter point is of special interest considering the nature of subliminal stimuli, which are processed by the brain, but not consciously perceived. Thus, we assume that automatic cognitive processes could be evoked, e.g. attentional, appraisal and implicit memory processes.

### Hypotheses

Based on the case of a heterosexual exclusively pedophilic forensic inpatient, we want to examine the potential of the eye tracking method and the fMRI measure to explore the impact of testosterone suppression to castration level on automatic and controlled attentional processes. According to the above discussed aspects we expected the following: (1) Eye tracking: (a) Based on the results of earlier studies we expected that the patient will show shorter fixation latency and longer relative fixation times to images of girls compared to images of women. (b) If ADT lowers the sexual motivation, longer fixation latencies and shorter relative fixation time to images of girls are presumed. (2) fMRI: (a) ADT should decrease hemodynamic responses to the subliminally presented sexual stimuli in brain regions which are associated with all four components of sexual arousal. But according to the characteristics of this kind of treatment the mentioned decrease should be rather unspecific to all kind of sexual stimuli than stimulus-specific. If subliminally presented arousal inducing stimuli activate (implicit) memory processes, as proposed by Brooks, Savov, Allzén, Benedict, Fredriksson and Schiöth [56], activation in hippocampal regions should be seen as well. (b) Furthermore, we questioned if the antiandrogen treatment leads to an increased activation in brain regions associated with inhibitory aspects of sexual arousal, representing a devaluation of sexual stimuli and inhibitory control processes. This should be seen in stronger hemodynamic responses in the left lateral and medial orbitofrontal cortex and in the lateral temporal cortex.

## Case presentation

The 47 year old patient was born as the youngest of five children. His father died shortly after his birth. He reported to be no victim of physical or sexual abuse in childhood or adolescence. He attended a school for mentally handicapped children without graduation. First criminal offences (burglary and sexual child abuse) occurred at the age of 14. Until now he was repeatedly convicted, mainly for burglary (at least 11 cases in the criminal records). Furthermore, he sexually abused at least 17 female child victims. Only the age of one victim (five years) is documented in the forensic records, but the patient himself reported victims aged between six – nine years. The criminal offences and his sexual deviance and dissocial personality disorder lead to several incarcerations in forensic high-security hospitals with rather short periods of probation since his 18^th^ year of life.

The patient fulfilled the diagnostic criteria for pedophilia [exclusive type, sexually attracted to females; DSM-IV 302.2; 1] and for a antisocial personality disorder (DSM-IV 301.7). The Psychopathy Checklist (PCL-R); [63] revealed a score of 29. Thus, he reached a cut-off value for psychopathy, which is common in European countries [64]. According to a recently conducted intelligence test he reached an Intelligence Quotient (IQ) of 98 (Wechsler Abbreviate Adult Intelligence Scale (WAIS) in the German version); [65]. Structural magnetic resonance imaging revealed no pathological findings. Sexual orientation assessed with the Kinsey Scale [66] showed in accordance with exclusively female child victims an exclusively heterosexual orientation. On the Screening Scale for Pedophilic Interests (SSPI) the patient reached a *Md* = 3, which identified a moderately high risk for recidivism [67,68]. The german version of the Bumby Molest Scale [69] counted a sum score of 114, revealing strong cognitive distortions in regard to sexual abuse of children. (about 2 SD over the mean of a control sample of pedophilic child molesters, [69]). The patient is at moderate risk for sexual recidivism according to the prognostic instrument Sexual Violence Risk - 20 in the German version [70].

Forensic hospital facilities have provided psychotherapy during the patient’s detention implementing treatment as usual. Many years ago, he received specific psychological group therapy according to standardized manuals addressing the treatment of sexual offenders. Based on the patient´s perspective, sexual fantasies and sexually deviant behavior was described excessively and in detail. Despite this long-term cognitive-behavioral treatment no victim empathy was noticeable. He did not realize the severity of harm he has done to the victims and was not able to eliminate any possibility of committing another sexual abuse.

His diagnosed antisocial personality disorder entailing a lack of empathy for victims, incapability to conform to social norms as well as the ineffective therapeutic treatment and the persistent pedophilic interest facilitate a high risk for recidivism. According to ADT recommendations of at least one forensic psychiatric evaluation, the antiandrogen medication with triptorelin (gonadotropin-releasing hormone agonist, Salvacyl®, 11.25 mg every 3 months) was initiated. Additionally, vitamin D (Calcivit D forte®) was given once on a daily basis in order to decrease the possible risk of osteoporosis. He did not receive any antiandrogens before the current treatment. According to respective recommendations the patient receives weekly supportive psychotherapy. The serum testosterone was measured before, four weeks later and five months after the onset of the antiandrogen medication. Serum testosterone levels were constantly suppressed during the treatment (before treatment: 5.89 μg/l, four weeks after start of treatment: 0.19 μg/l, five months after start of treatment: 0.23 μg/l). Before treatment onset the patient stated that he masturbated once a month with pedophilic sexual fantasies. Thereby, he reported an increase in masturbation frequency after the onset of the treatment in order to test his physical functioning.

After three months of treatment, the patient was not able to masturbate anymore due to loss of physical functioning. He reported sexual and deviant fantasies as absent. But, considering his detention in a locked forensic psychiatry unit, the patient might have realized the consequences of having and reporting intensive sexual fantasies and needs during therapy which could have been interpreted as an indicator for higher risk of relapse committing sexual abuse from the perspective of a therapist.

### Methods

#### Eye tracking measures

**Figure 1:**
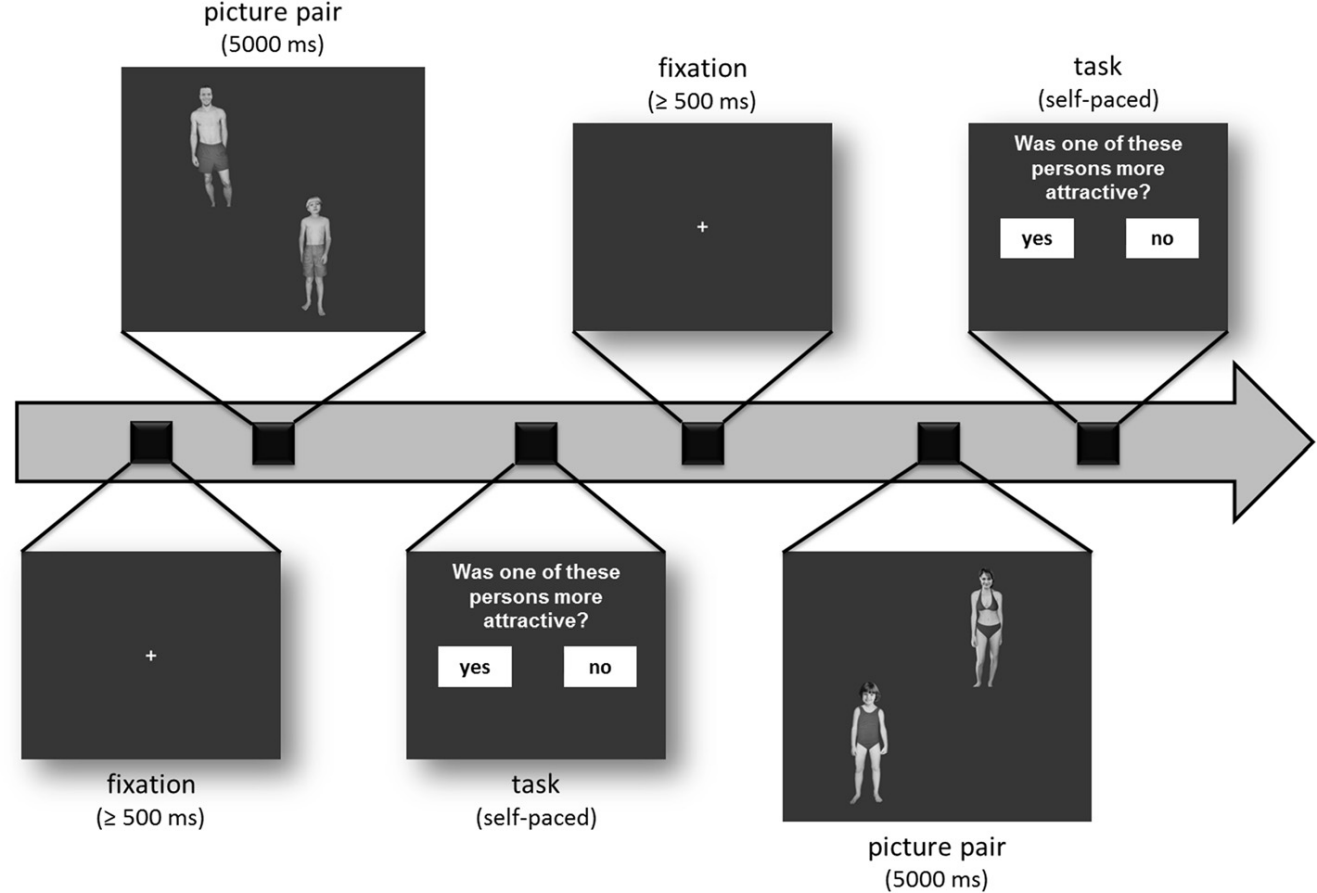
**Illustration of the experimental design in the eye-tracking experiment.** Note that these sample images were not among the experimental stimuli.

Eye tracking measures were conducted three weeks before initial antiandrogen medication and 4 months after the onset of ADT. Figure 1 displays the experimental design for the eye tracking measurements. Throughout the experiment (64 trials) eye movements were recorded. Before each trial, a fixation cross (approximately 1° x 1°) appeared at the center of the screen. Subsequently, two images appeared and remained for 5000 ms. After each stimulus presentation a question appeared (“Was one of these persons sexually more attractive?”) and the participant had to respond using the computer mouse. This task was introduced to distract the participant from eye movement measures and had no content related meaning.

Each stimulus display showed two images that were presented in two opposing corners of the computer screen (see Figure 1 for an example). The locations of the images were randomly balanced across trials. Two different stimulus-combinations existed: either the image of a girl combined with the image of a woman or the image of a boy combined with the image of a man. All stimuli were derived from the Not-Real-People (NRP) picture set [71,72]. The NRP-set is a stimulus set that does not display images of real people and meets contemporary legal and ethical requirements [73]. The images were non-pornographic, depicting solely non-explicitly sexual poses or sexual activities. In this study, only male and female nude persons from the Tanner stages 1 and 2 (as category “child”), 4 and 5 (as category “adult”) were used, four from each stage [74].

Eye movements were measured using a SMI iView X™ RED eye tracker (SensoMotoric Instruments GmBH, Berlin, Germany) in combination with an iView X™ workstation by measuring the corneal reflection and dark pupil with a video-based infrared eye camera. The participants were seated in a quiet room facing the monitor at eye level at a viewing distance of 27.6 inches in front of the monitor. In order to identify fixations and saccades, the raw eye movement data were basically analyzed with BeGaze™3 (SensoMotoric Instruments GmbH, Berlin, Germany). Statistical analysis was performed with SAS 9.2 (SAS Institute Inc., Cary, NC, USA). Further details of data analysis are described in Additional file 1 (see 2. Eye tracking: Data analysis).

#### FMRI-measures

**Figure 2:**
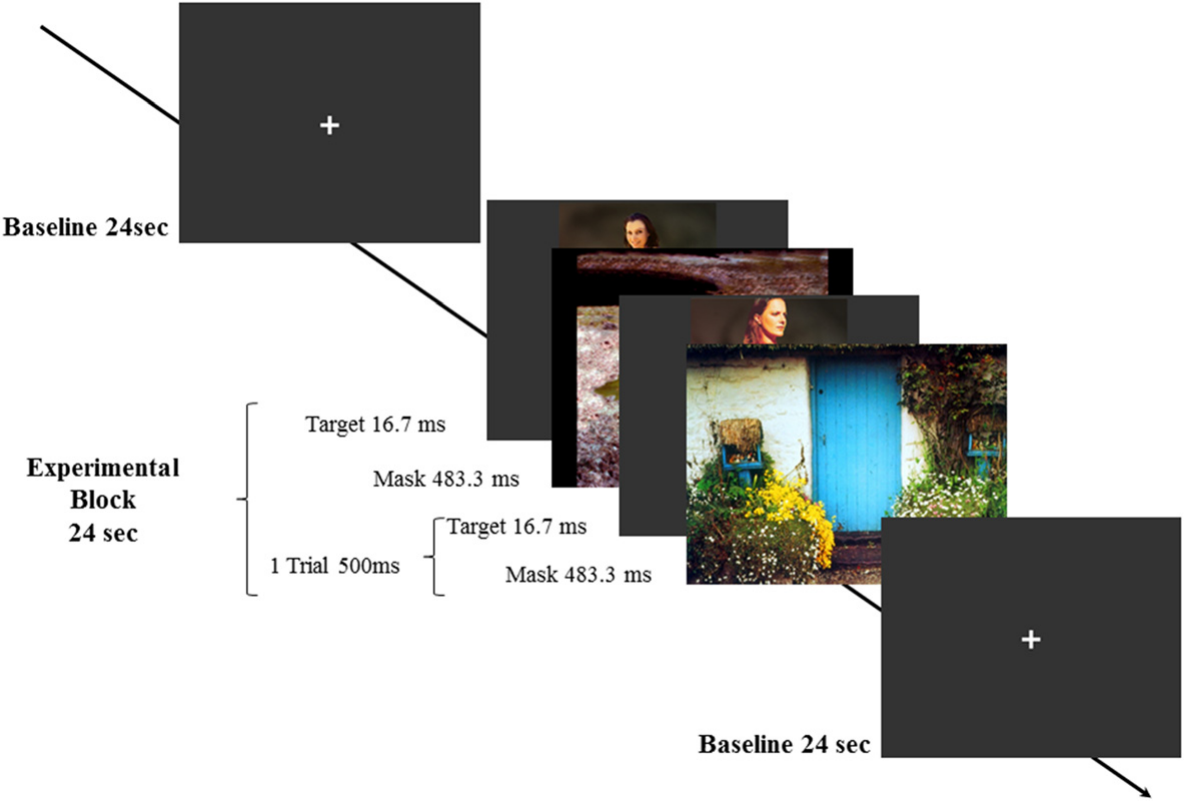
**Illustration of the experimental design in the fMRI-experiment.** Presented is an example for one experimental block containing subliminal presented sexual images and mask images and the baseline condition with a duration of 24 sec.

FMRI measures were conducted two months before initial antiandrogen medication and four months after the onset of ADT. The patient was told that different images will be presented and he will be asked afterwards to rate the presented images according to different qualities (sexual arousal, valence). Stimuli were presented in a block-design (Figure 2) with experimental blocks presenting the stimuli of one category and baseline blocks presenting a fixation cross. Each block lasted 24 sec. One experimental block consisted of 48 trials, each lasting 500 ms. One trial matches to a backward-masking design containing one target stimulus immediately followed by one mask stimulus. Target stimuli were subliminally presented for 16.7 ms, the mask stimulus was shown for 483.3 ms, followed by the next target stimulus. Target stimuli were the same stimuli as used in the eye tracking task: the NRP-picture set with the categories girl, boy, woman and man, but in the colored version. Neutral masking stimuli (with low arousal and average valence score) were selected from the IAPS-set (International Affective Picture Set [75]. A detailed list of all IAPS-images including valence and arousal values can be found in the Additional file 1 (see 3. IAPS-stimuli, Table S1). Overall, 16 target stimuli of each category were presented in a randomized order, each thrice.

After the experiment the patient had to solve a recognition task. All presented target and mask stimuli as well as the same number of new target and mask stimuli were shown. He was asked to determine if he had seen the presented images during the fMRI-session or not.

The MRI scans were acquired at 3 Tesla (Siemens Magnetom TIM Trio, Siemens Healthcare, Erlangen, Germany) with an 8-channel phased-array head coil. For anatomical reference, a three-dimensional (3D) T1-weighted dataset was acquired (MPRAGE: Magnetization Prepared Rapid Acquisition Gradient Echo; echo time: 3.26 ms; repetition time: 2250 ms; inversion time: 900 ms, flip angle 12°; 176 slices; isotropic resolution of 1 × 1 × 1 mm^3^). The functional datasets were acquired using 2D gradient-echo echo-planar imaging (echo time: 30 ms, repetition time: 2000 ms, flip angle: 70°, voxel size: 3 × 3 × 3.0 mm^3^, 33 slices). Two sessions were conducted with duration of about 15 min each. A total of 433 whole brain volumes were recorded within one session. Identical MRI-protocols were applied in the pre- and the post-measurement. Image analysis was performed using Statistical Parametric Mapping (SPM8, Wellcome Trust Center for Neuroimaging, http://www.fil.ion.ucl.ac.uk/spm/). Further details of data analysis are described in the Additional file 1 (see 4. fMRI: Data analysis).

#### Stimulus rating

Subsequent to the experimental phase, the patient rated all target and all masking stimuli in respect to valence and sexual arousal on a 9-point Likert scale (1 = unpleasant/not arousing, 9 = pleasant/arousing). In order to additionally assess viewing time, the interval between the stimulus onset and the completion of the second rating (sexual arousal rating) was measured, without patient´s knowledge.

## Results

### Stimulus rating

The patient viewed images of girls longer than images of women before and after four months of ADT. This difference was significant in the pretest (girl: 4822.11 ms ± 1944.40 (mean ± standard deviation), woman: 2514.44 ms ± 1170.34, t(24.6) = 4.07, p < .0001). In the posttest the difference was smaller and non-significant, due to a decrease of viewing time to girls and a slight increase of viewing time to women (girl: 3925.04 ms ± 2997.61, woman: 2931.49 ms ± 1366.40, t(20.98) = 1.21, p = .241). The viewing time data was also analyzed separately for the images of girls and women (comparing pre- and posttest). No significant changes were detectable during ADT (girl: t(15) = 1.02, p = .32, woman: t(15) = -1.08, p = .3). The viewing time for the masking stimuli was 1884.59 ms ± 508.86 before and 3359.42 ms ± 2776.10 after four months of treatment. The patient rated all stimuli for valence and sexual arousal between 1 and 2 (median) of the 9-point Likert scale, except the images of women in the posttest (5 at the 9-point Likert scale for the valence).

### Eye tracking

**Figure 3:**
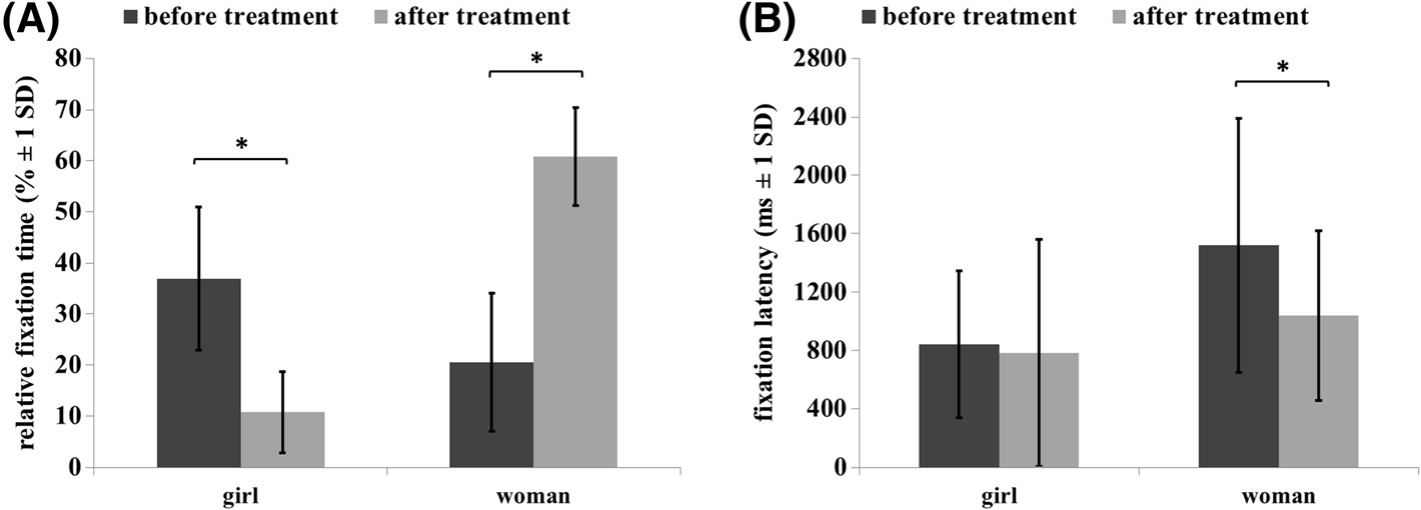
**Eye tracking measures.** Mean of relative fixation time and fixation latency for the patient before and after 4 months antiandrogen treatment.

Before ADT, the patient showed a significantly higher relative fixation time for images of girls compared to the images of women (t(62) = 4.76, p < .0001). After four months of ADT, this result was reversed (t(62) = -22.74, p < .0001). The statistical analysis separately for images of girls yielded a significant decrease of the relative fixation time after four months of ADT (t(31) = 8.99, p < .0001). In contrast, the relative fixation time for images of women showed a significant increase after four months of ADT (t(31) = -14.18, p < .0001) (see Figure 3a).

In regard to automatic attentional processes, before treatment the patient showed a significantly lower fixation latency for images of girls compared to images of women (t(49,64) = -3.83, p < .0001). After treatment, the fixation latency was likewise lower for girls than for women, but this difference was not significant (t(55) = -1.43, p = .157). This should be attributed to a decrease of the fixation latency for images of women. The direct comparison of the fixation latency for girls before and after treatment demonstrated no significant changes of fixation latency after four months of ADT (t(24) = -.046, p = .96). On the contrary, the analysis revealed a significant decrease of fixation latency for women after four months of ADT (t(31) = 3.36, p < .01) (see Figure 3b).

### Recognition test

After the fMRI sessions, a recognition test was applied in order to assure the subliminal presentation of the target stimuli. The patient did not recognize any of the subliminally presented target stimuli whether before nor after four months of ADT (correct recognition rate: 0%) and correctly rejected all of the new presented images as new (correct rejection rate: 100%). In contrast, he correctly recognized 83.3% of the supraliminally presented masking stimuli in the pre- and the posttest. The correct rejection rate for newly presented mask stimuli was 94.4% in the pretest and 100% in the posttest. Thus, the recognition test demonstrated the successful subliminal presentation of target stimuli.

### FMRI-results

#### Comparison of hemodynamic responses to images of girls before and after four months of antiandrogen treatment

**Figure 4:**
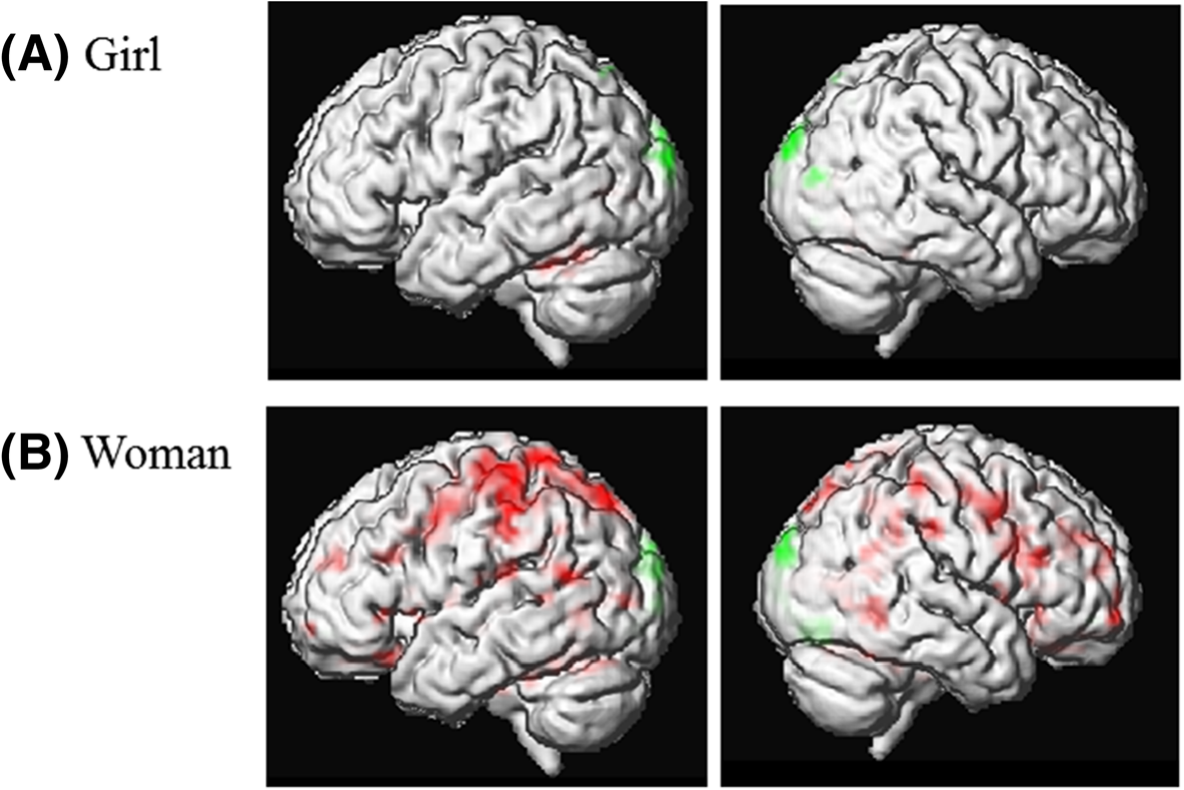
**fMRI measures.** Areas of significant hemodynamic responses obtained for the contrast comparing images of girls between the pre- and the posttest **(A)** and images of women between the pre- and the posttest **(B)**. The activation is superimposed on the individual brain of the patient. Statistical significance threshold: p ≤ .01 (uncorr.), spatial extend: 5 voxel. A masking procedure was applied, masking each comparison with the appropriate baseline contrast at p ≤ .05 (uncorr.) (for details see Additional file 1, 4. fMRI: data analysis). Red: Pretest > Posttest. Green: Posttest > Pretest.

**Figure 5:**
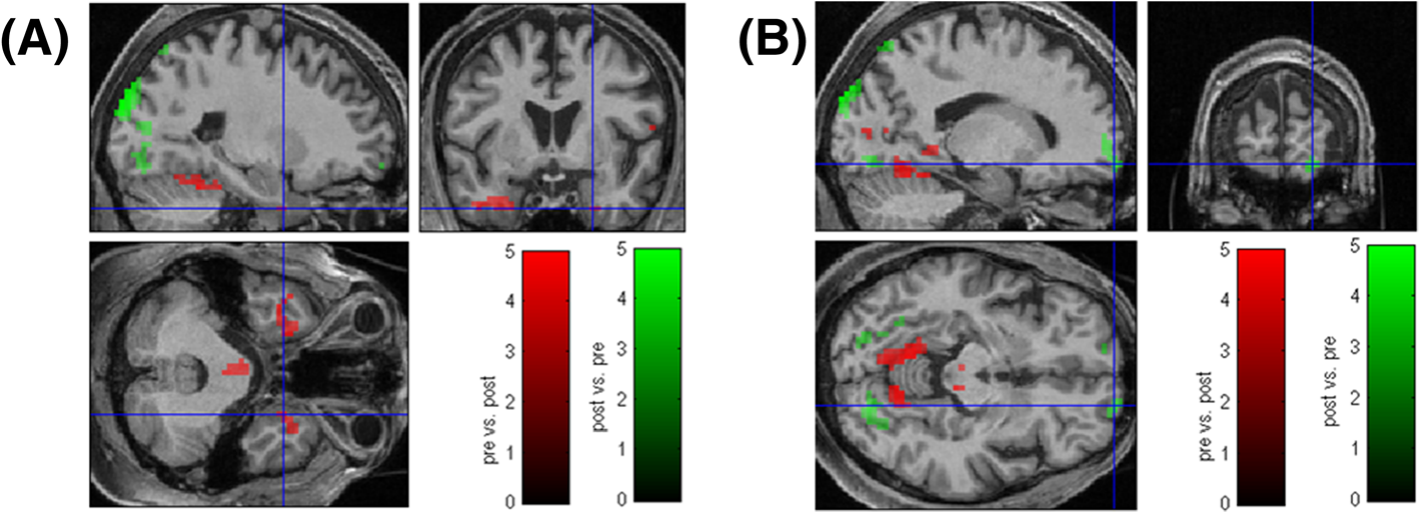
**fMRI measures.** Areas of significant hemodynamic responses obtained for the contrast comparing images of girls between the pre- and the posttest. The activation is superimposed on the individual brain of the patient. Statistical significance threshold: p ≤ .05 (uncorr.), spatial extend: 5 voxel. A masking procedure was applied, masking each comparison with the appropriate baseline contrast at p ≤ .05 (uncorr.) (for details see Additional file 1, 4. fMRI: data analysis). **(A)** Activation cluster in the right Hippocampus (entorhinal cortex), cytoarchitectonic probability: 90%, MNI-coordinates of the local maximum: x = 24 mm, y = 3 mm, z = -37 mm. **(B)** Activation cluster in the right middle orbital gyrus, MNI-coordinates of the local maximum: x = 16 mm, y = 64 mm, z = -12 mm. Red: Pretest > Posttest. Green: Posttest > Pretest.

The subliminal presentation of girls during the pretest revealed stronger activation in the following brain regions compared to the posttest: bilateral cerebellum, bilateral fusiform, lingual and calcarine gyrus and the left inferior temporal gyrus. Given this specific case of a pedophilic patient we were especially interested in the changes of hemodynamic responses to the images of girls. Therefore, we additionally used a more liberal significance threshold in order to screen if other brain regions showed changes after four months of ADT. Lowering the threshold to p < .05 (uncorr.) additional activation in the left amygdala/hippocampus region and in the right hippocampus was detectable. According to the probabilistic cytoarchitectonic maps [76] these activation clusters originated from the hippocampus. For the opposite comparison, i.e. which brain regions were more activated in the post- compared to the pretest, we found stronger activation in the posttest in the bilateral superior and middle occipital gyrus, the superior parietal lobule, and in the right lingual gyrus and precuneus. Lowering the threshold to p < .05 the bilateral superior and right middle orbital gyri were found to be activated (see Figures 4 and 5, and Additional file 1: Table S3).

#### Comparison of the hemodynamic responses to images of women before and after four months of antiandrogen treatment

The subliminal presentation of women during the pretest compared to the posttest revealed widespread stronger activation in the pretest in bilateral frontal, orbital, temporal, parietal and occipital regions, the cingulate cortex, the cerebellum, thalamus and insula. The posttest showed stronger activation in the bilateral middle and superior occipital gyrus, bilateral calcarine gyrus, the right cuneus, right fusiform gyrus and right medial orbital gyrus. Lowering the threshold to p < .05 additional activation was detectable in the left anterior cingulate cortex (see Figure 4, and Additional file 1: Table S4).

## Discussion

In this study we compared the data of a heterosexual exclusively pedophilic patient before and after four months of antiandrogen treatment. Before discussing the results we want to point out that this is a single case study. The accomplished results and interpretations cannot be generalized. But they can serve as a first impression of what we could expect in further studies with larger groups of subjects.

Based on the eye tracking results it seems that ADT affects only controlled but not automatic attentional processes while viewing sexually relevant stimuli. The subliminal presentation of sexually relevant stimuli measured with fMRI showed that ADT leads to changed activation patterns in occipital and parietal brain regions, the hippocampus and in the orbitofrontal cortex. A decrease of activation in regions associated with the perceptual and implicit memory processes is accompanied by an increase in brain areas linked with attentional processes, inhibition and the devaluation of the stimuli. Our suggestion is that ADT induces changes in the processing of sexually relevant stimuli, also at an unconscious level, reflecting additional changes of perceptive and cognitive automatic processes.

### Eye tracking

In regard to controlled attentional processes, the results differed between pre- and posttest. Before treatment, the patient was more attracted to images of girls compared to images of women. After treatment, he was more attracted to women than to girls. In contrast, concerning automatic attentional processes, the eye-tracking method demonstrated that the ADT did not affect the initial orienting reaction on sexually deviant stimuli: Before and after treatment the patient showed lower fixation latency for images of girls than for images of women. Our previous studies demonstrated fixation latency as a parameter for automatic attentional processes representing the individual sexual preference [36,37]. We conclude that the antiandrogen medication had no impact on the pedophilic preference of the patient. Therefore, the eye tracking method might uncover variables which are stable under ADT and variables which are influenced by the ADT.

The relative fixation time reflects controlled attentional processes and showed changes between the first and second eye tracking assessment. One possible explanation for this finding is that the changes in relative fixation time are an effect of the antiandrogen medication: the ADT may have enhanced the ability of the patient to avoid deviant sexual stimuli or lowered the need to look at the deviant but preferred sexual stimuli. Based on the assumption that the antiandrogen medication decreased the (overall) sexual drive of the patient [7,77], it could be speculated, that under medication he was able to consciously control the time that he was looking at the sexual images, i.e. the fixation time. Another possible explanation is that the patient actively tried to manipulate the test result by responding in a socially desirable manner. Given his well-known dissocial personality disorder, his familiarity with the experimental design and his willingness to leave the hospital as soon as possible (and by himself), the mentioned reasons seem quite conceivable. Moreover, it seems to be obvious for the patient to assume that the goal of the eye tracking is to explore where the subjects are looking at. Since the patient has five seconds to control his eye movements, the relative fixation time is consciously influenceable [34]. At first glance these changes in relative fixation time seem to be in contrast to the unchanged viewing time. But it has to be taken into account that in contrast to the relative fixation time, viewing time is measured without the knowledge of the subject while rating stimuli [73]. Thus, the chance to manipulate the viewing time variable is relatively low.

On the contrary, the fixation latency reflects early automatic attentional processes. Thus, it should be difficult to manipulate this variable [78,79]. The results for this variable before and after the treatment remained stable, i.e. the shorter fixation latency for the images of girls compared to the images of women. But this difference was smaller in the posttest. This could be an indication of the patient trying to manipulate his eye movements in the very beginning of the trial, but failed in contrast to the putative manipulations of the relative fixation time. Nevertheless, the results concerning the fixation latency demonstrated that lowering the testosterone level to castration level has no impact on the deviant sexual orientation. Furthermore, the unchanged fixation latency demonstrated that even under antiandrogen medication an initial orienting reaction to pedophilic stimuli can occur. Nevertheless, our hypothesis suggesting an overall increase of the fixation latency due to decreased sexual motivation does not match with the achieved results after all. Here it could be argued that the parameter is resistant against the influence of sexual motivation. According to us, fixation latency is rather modulated by the sexual preference of the subject.

In summary, risk assessment and prognosis could benefit from this acquired information pointing out the importance of concomitant psychotherapy which itself entails risk management.

### Functional imaging

ADT lead to decreased hemodynamic responses to the subliminally presented images of girls in brain regions mostly associated with visual perception, i.e. the calcarine and lingual gyrus, the fusiform gyrus and the inferior temporal gyrus. On the one hand the decreased brain activation after four months of ADT could reflect unspecific habituation processes in those areas, as it has been described for the repeated presentations of subliminal stimuli [56,80]. On the other hand it has also been discussed that lateral brain areas, i.e. the fusiform gyrus and the inferior temporal gyrus do not only respond to low level features of the visually presented stimuli such as color or luminance but also to the general emotional salience and more specifically to the sexually arousing character of the presented images [38]. Therefore, the decreased activation in the fusiform gyrus and the inferior temporal gyrus could reflect the decreased sexually arousing character of the girls for the patient after four months of ADT. A positive association was found between the testosterone concentration and hemodynamic responses in the inferior temporal cortex in healthy subjects while they viewed erotic stimuli [81] which confirms our results. At a more liberal significance threshold a decrease of hemodynamic responses in the bilateral hippocampal region was observed. This might result from decreased implicit memory processes indicating a decreased reactivity of the patient while viewing the images of girls during ADT. As mentioned above, the hippocampus is one of the brain regions containing androgen receptors. After castration a significant decrease of the CA1 (cornu ammonis) spine synapse density in the hippocampus of male nonhuman primates has been described [82].

In summary, our study partially supports the results of the three single case fMRI-studies, examining the effect of ADT in pedophilic patients. Similar to Moulier et al. and Schiffer et al. we found a decrease of hemodynamic responses in brain regions linked with perceptual processes and in the hippocampal region [53,54]. Another interesting point concerns the amygdala. The above discussed activation was assigned to the hippocampal region. But the cluster probably also comprised parts of the amygdala. The amygdala, as a region associated with emotional valuation, arousal and valence, has often been examined with respect to a pedophilic sexual interest. A reduced right amygdalar volume was found in pedophiles compared to controls [83]. In another study pedophilic patients exhibited a stronger response to sexually relevant stimuli in the amygdala compared to controls [45]. As already mentioned in the introduction, a decrease of amygdala activation was seen under ADT in a single case study with a pedophilic patient [55]. Further research has to be done to examine if changes under ADT occur in hippocampal and/or amygdalar regions.

To our knowledge none of the studies examined which brain regions showed stronger activation under ADT compared to the pre-treatment condition. In contrast to our hypotheses, under ADT the patient showed stronger hemodynamic responses to images of girls in the bilateral superior and middle occipital gyrus, in the superior parietal lobule, and in the precuneus. According to the four-component model of sexual arousal parietal regions are associated with cognitive/attentional processes. Linking this activation pattern with the expected increased activation in the bilateral superior and right middle orbital gyri, it could be assumed that under ADT the patient’s attentional processing of the images of girls is associated with more intensive appraisal processes as well as with inhibitory control processes – even under unconscious conditions. Taking these results together with the accomplished eye tracking data, it could be speculated that the activated brain regions associated with attentional processes and appraisal, represent the stable pedophilic interest of the patient, demonstrated with the invariant fixation latency as an indicator of automatic processes. Likewise, the stronger activation in brain regions associated with inhibitory control could be associated with the decreased relative fixation time in response to images of girls. This might indicate a more serious attempt on the part of the patient in order to control his own behavior.

The subliminally presented images of women elicited a rather unspecific decrease in hemodynamic responses in various brain regions under the influence of ADT. Besides the above mentioned unspecific habituation processes to repeatedly presented subliminal stimuli, the decreased hemodynamic responses could be a reflection of lower common reactivity of the patient to sexually salient stimuli. Considered the effect of ADT, i.e. lowering the testosterone concentration to castration level it is comprehensible that not only the deviant sexual interest but rather the general sexual drive of the patient should be diminished.

The increased activation in higher visual areas and the medial orbital gyrus similarly to the evoked responses to images of girls could reflect attentional processes and the devaluation of the stimuli. This corresponds to the eye tracking results demonstrating that the patient looked longer at images of women after ADT and, as already discussed above, could imply a better ability to control the own behavior and to answer in a socially desirable manner. In contrast, the stronger activation in the fusiform gyrus (and also in primary visual regions) could be associated either with the processing of low level features of the visually presented stimuli such as color or luminance or with the general emotional salience and more specifically with the sexually arousing character of the presented images. Furthermore, under ADT the patient reported not having any sexual or deviant fantasies. This corresponds to the stronger activation in brain regions linked with inhibitory processes and the devaluation of the stimuli.

## Conclusions

In the case of a pedophilic forensic inpatient we explored the potential of the eye tracking method and a new fMRI-design for the evaluation of the therapeutic outcome of ADT. With the eye tracking method we showed that controlled attentional processes could change under antiandrogen medication, whereas automatic processes remained mostly stable. We assume that these results reflect either the increased ability of the patient to control his eye movements while viewing sexually preferred stimuli or his competence to answer in a socially desirable manner. Furthermore, unchanged automatic attentional processes might reflect the stable pedophilic preference of the patient. In the fMRI-study the subliminal presentation of visual sexual stimuli showed that even at an unconscious level ADT leads to changes in processing sexually relevant stimuli in the patient, supposedly reflecting changes in cognitive and perceptive automatic processes. A decrease of activation in regions associated with perceptual processing and implicit memory processes was accompanied by an increase in brain areas linked with attentional processes, inhibition and the devaluation of the stimuli. Thus, while the eye tracking study revealed a stable pedophilic preference of the patient under ADT based on automatic attentional processes, the results of the fMRI-study indicate that the automatic processing of pedophilic stimuli might change under ADT as well. Taking the restricted significance of a single case study into consideration, these first results are preliminary and further research is necessary to differentiate these automatic processes in more detail.

However, it is important to note, that eye tracking and fMRI alone are not able to show all relevant therapeutic effects of an antiandrogen treatment. According to current knowledge about the effects of testosterone and ADT a lot of aspects should be considered, e.g. physiological, endocrinological, sexual, neurophysiological and psychological aspects [3]. Moreover, further studies with larger groups of healthy subjects and patients have to be conducted to verify or falsify the results of our single case study. If these measures can reliably deliver additional and significant information, they could be integrated into evaluation studies of ADT prospectively.

## Consent

The study was approved by the ethics committee of the medical faculty of the Georg-August-University of Göttingen. The patient was carefully informed of the possible effects and side effects of the antiandrogen medication as well as possible consequences of the eye tracking method and the fMRI measures. We paid special attention to the potentially problematic situation of his status as an inpatient. The patient was briefed in detail orally as well as in written form about the fact that neither his agreement nor his denial to participate in the study would lead to positive or negative consequences, sanctions, advantages or disadvantages for him. Furthermore, he was informed that anytime he could end his participation in the study without any justification and negative consequences or disadvantages. The study information sheet and informed consent contained a paragraph with these remarks.

He gave his written informed consent to participate in the study, for the publication of his data and further clinical details.

## Competing interests

The authors declare that they have no competing interests.

## Authors’ contributions

KJ has been involved in the development of the design, data analysis and the preparation of the manuscript. PF has made important contributions to the development and programming of the design, data collection, data analysis and the intensive revision of the manuscript. HL gave substantial clinical support, supervised the clinical tests and was involved in drafting and revision of the manuscript. PD supervised the fMRI-measurement and was involved in drafting and revision of the manuscript. JLM supervised and supported the development of the idea and the design and was involved in the drafting and revision of the manuscript. All authors have read and approved the final manuscript.

## Pre-publication history

The pre-publication history for this paper can be accessed here:

http://www.biomedcentral.com/1471-244X/14/142/prepub

## Supplementary Material

Additional file 1: Figure S1 The four-component model of sexual arousal. **Table S1.** presents the neutral masking stimuli selected from the IAPS-set (International Affective Picture Set). **Table S2.** Statistical analysis of the fMRI-data. **Table S3.** Brain areas and stereotaxic coordinates for the subliminally presented images of girls. **Table S4.** Brain areas and stereotaxic coordinates for the subliminally presented images of women. Click here for file
