# Supplementary material for: Changed processing of visual sexual stimuli under GnRH-therapy – a single case study in pedophilia using eye tracking and fMRI
Source: BMC Psychiatry. 2014 May 17;14:142. doi: 10.1186/1471-244X-14-142 (PMC4036749; doi:10.1186/1471-244X-14-142)
Supplement: Additional file 1: Figure S1 — The four-component model of sexual arousal. Table S1. presents the neutral masking stimuli selected from the IAPS-set (International Affective Picture Set). Table S2. Statistical analysis of the fMRI-data. Table S3. Brain areas and stereotaxic coordinates for the subliminally presented images of girls. Table S4. Brain areas and stereotaxic coordinates for the subliminally presented images of women. [file 1471-244X-14-142-S1.doc]

**Changed processing of visual sexual stimuli under GnRH-therapy –**

**A single case study in pedophila using eye tracking and fMRI**

**Jordan/Fromberger et al.**

**Supplementary Material**

**1. Introduction: The four-component model**

**
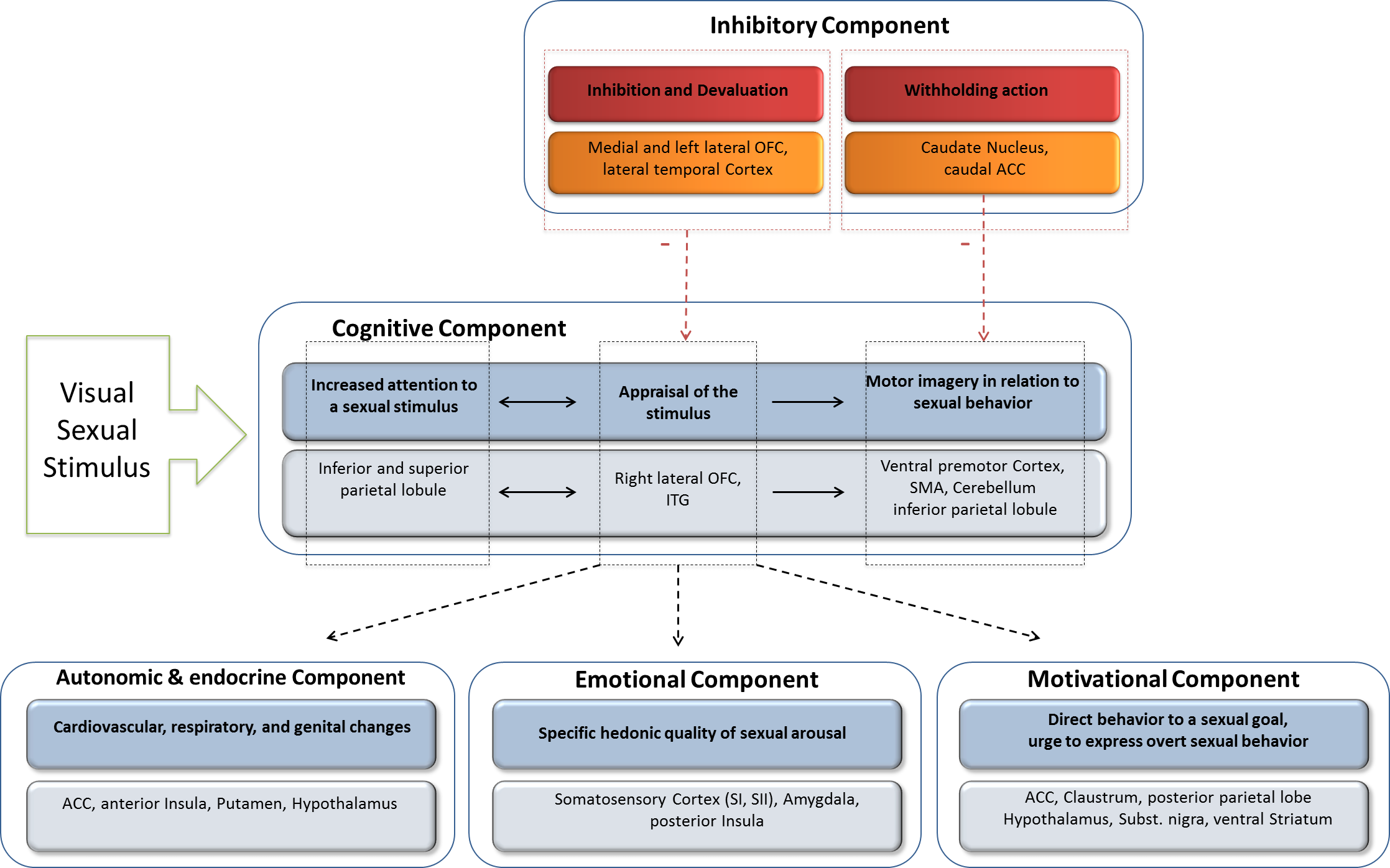
**

Figure S1. The four-component model of sexual arousal . Modified and translated with permission from .

ACC: Anterior cingulate cortex, CB: Cerebellum, ITG: Inferior temporal cortex, OFC: orbitofrontal cortex, SMA: supplementary motor area Blue: excitatory components, phenomenological level

light blue: excitatory components, neuronal level

red: inhibitory components, phenomenological level

orange: inhibitory components, neuronal level

**2. Eye tracking: Data analysis**

In order to identify fixations and saccades, the raw eye movement data were basically analyzed with BeGaze^TM^3 (SensoMotoric Instruments GmbH, Berlin, Germany). In order to analyze visual attention directed towards different aspects of the stimulus display, we divided each stimulus display into two areas of interest (AOI). Every image (woman, man, girl, boy) equates to one AOI. Two eye movement variables were measured. Relative fixation time was defined as the sum of fixation-duration of all fixations located in the space of the relevant AOI, divided by the whole presentation time. Relative fixation time is a measure for the overall attention a specific AOI attracts . In contrast, fixation latency reflects early attentional processes. Fixation latency was defined as the duration from the stimulus onset to the first fixation within an AOI, representing a measure for initial orientation (for further details regarding the experimental design see .

**3. IAPS-stimuli**

Table S1 presents the neutral masking stimuli selected from the IAPS-set (International Affective Picture Set). Picture-number, mean (M) and standard deviation (SD) for the ratings of valence and arousal (male subjects, 9-point likert scale: 1= negative valence/low arousal, 9= positive valence/high arousal) are given .

| **Description** | **Picture No.** | **Valence** | | **Arousal** |  |
| --- | --- | --- | --- | --- | --- |
|  |  | **M** | **SD** | **M** | **SD** |
| Flowers | 5731 | 5.19 | 1.62 | 2.44 | 1.87 |
| Plant | 5740 | 5.07 | 1.27 | 2.36 | 1.77 |
| Spoon | 7004 | 4.89 | 0.6 | 2.09 | 1.75 |
| Bowl | 7006 | 4.65 | 1.1 | 2.08 | 1.58 |
| Basket | 7010 | 4.95 | 1.43 | 1.55 | 1.36 |
| Fan | 7020 | 5.02 | 1.22 | 2.15 | 1.71 |
| Mug | 7035 | 4.81 | 1.05 | 2.56 | 1.8 |
| DustPan | 7040 | 4.72 | 1.19 | 2.46 | 1.86 |
| Fork | 7080 | 5.43 | 1.26 | 1.98 | 1.63 |
| Book | 7090 | 4.95 | 1.54 | 2.3 | 1.9 |
| Hammer | 7110 | 4.51 | 1.02 | 1.91 | 1.39 |
| Lamp | 7175 | 4.78 | 1.18 | 1.55 | 0.96 |
| AbstractArt | 7185 | 4.84 | 1.07 | 2.56 | 2.13 |
| AbstractArt | 7187 | 4.87 | 1.12 | 2.16 | 1.63 |
| ClothesRack | 7217 | 4.63 | 1.15 | 2.31 | 1.64 |
| Plate | 7233 | 5.01 | 1.21 | 2.51 | 1.74 |
| Cabinet | 7705 | 4.75 | 0.79 | 2.4 | 1.78 |
| Tissue | 7950 | 4.62 | 1.26 | 2.3 | 1.89 |

**4. FMRI: Data analysis**

Image analysis was performed using Statistical Parametric Mapping (SPM8, Wellcome Trust Center for Neuroimaging, <http://www.fil.ion.ucl.ac.uk/spm/>). The same preprocessing steps were applied separately for pre- and posttest. Functional images were corrected for motion artifacts. The structural image of the patient was coregistered to his mean functional image. The automated segmentation algorithm provided by SPM8 was applied for segmentation and normalization of the structural image. Functional images were normalized into a common stereotaxic reference space (International Consortium for Brain Mapping: http://www.loni.ucla.edu/ICBM/) using the normalization parameters determined by the segmentation of the structural image (isotropic voxel= 3.2*3.2*3.2mm). Spatial smoothing was applied using a 7 mm full-width at half-maximum Gaussian kernel. For statistical analysis, a combined design matrix was generated for the pre- and posttest conditions.

A general linear model (GLM) was built separately for each session with a fixed effect approach, containing the regressors of interest, e.g. the different target stimuli categories as well as one for each of the six rigid-body movement parameters determined by the realignment procedure. The resulting set of voxel values for each contrast constitutes a statistical parametric map of the T-statistic (SPM(T)). According to our hypotheses we were mainly interested in changes in hemodynamic responses under antiandrogen therapy. Therefore, the contrasts of interest were the differential effects between the pre- and posttest of the images of girls and women separately. The statistical threshold was set to p<.01 (uncorrected for multiple comparisons) and a spatial extend of k=5 voxels. This liberal threshold was used according to the, as expected, small differences in hemodynamic responses of two types of the subliminally presented images and the special situation of one single case. For each condition (images of girls and women) we examined separately which brain regions showed a stronger activation in the pretest compared to the posttest and which brain regions showed a stronger activation in the posttest compared to the pretest. Therefore we applied one-sided t-tests (typical SPM T-contrasts) and used an inclusive masking procedure. Table 2 presents the four analyzed SPM T-contrasts as well as selected masking contrasts. In the following, this procedure will be described in more detail for one analyzed outcome of a comparison, namely: Which brain regions were stronger activated during the subliminal presentation of girls in the pretest compared to the subliminal presentation in the posttest, we computed the SPM T-contrast girl-pretest versus girl-posttest using the statistical threshold p<.01 (uncorrected for multiple conditions) and a spatial extend of k=5 voxels. In order to restrict our analysis to those brain regions activated in the pretest, we applied an inclusive spatial masking procedure, using the SPM T-contrast for the subliminal presentation of the girl images in the pretest (girl-pretest) as masking images with a statistical threshold of p<.05 (uncorrected for multiple comparisons). Voxels outside of the mask were excluded from the analysis. This procedure was applied for each of the four analyzed contrasts using the individual masking images (see Table 2).

Brain regions were identified using probabilistic cytoarchitectonic maps in the Anatomy Toolbox 1.8 implemented in SPM8 (<http://www.fz-juelich.de/inm/inm-1/DE/Forschung/_docs/SPMAnatomyToolbox/SPMAnatomyToolbox_node.html>) .

Table S2: Statistical analysis of the fMRI-data. Comparisons of interest, computed SPM T-contrasts and masking images.

| **Comparison of interest** | **computed T-contrast** | **Inclusive masking contrast** |
| --- | --- | --- |
| Images of girls: Stronger activation in the pretest compared to the posttest | Girl-pretest > Girl-posttest | Girl-pretest |
| Images of girls: Stronger activation in the posttest compared to the pretest | Girl-posttest > Girl-pretest | Girl-posttest |
| Images of women: Stronger activation in the pretest compared to the posttest | Woman-pretest > Woman-posttest | Woman-pretest |
| Images of women: Stronger activation in the posttest compared to the pretest | Woman-posttest > Woman-pretest | Woman-posttest |

**5. FMRI-results**

Table S3: Brain areas and stereotaxic coordinates for the subliminally presented images of girls.

| **Com-parison** | **Brain area** | **Cluster**  **Size**  **[voxel]** | **Anatomical region**  Cytoarchitectonic probability | **R/L** | **x y z** | **Significance**  **T** |
| --- | --- | --- | --- | --- | --- | --- |
| **Pretest**  **> Posttest** | Cerebellum/  Occipital lobe | 87 | Cerebellum | L | -24 -58 -24 | 3.43** |
|  |  |  | Fusiform gyrus | L | -36 -54 -21 | 3.42** |
|  |  |  | Lingual gyrus (BA18: 30%) | L | -17 -51 -5 | 2.71* |
|  |  | 7 | Cerebellum | R | 2 -64 -21 | 3.38 |
|  | Occipital lobe | 47 | Lingual gyrus (BA18: 40%) | R | 12 -64 -8 | 3.10** |
|  |  |  | Calcarine gyrus (BA17: 70%) | R | 8 -67 8 | 2.93* |
|  |  | 37 | Calcarine gyrus (BA17: 30%) | L | -24 -67 4 | 2.97* |
|  |  |  | Lingual gyrus (BA18: 60%) | L | -8 -80 1 | 2.83* |
|  |  |  | Calcarine gyrus (BA17: 80% | L | -1 -77 8 | 2.61* |
|  | Temporal lobe | 8 | Inferior temporal gyrus | L | -49 -48 -24 | 2.98* |
|  |  | 9 | Fusiform gyrus | R | 31 -35 -24 | 2.75* |
| **Posttest > Pretest** | Occipital lobe | 67 | Superior occipital gyrus  (BA18: 40%) | R | 24 -93 24 | 4.74** |
|  |  | 80 | Superior occipital gryus  (BA18: 30%) | L | -17 -99 20 | 3.98** |
|  |  |  | Superior occipital gyrus  (BA17: 60%) | L | -8 -96 11 | 3.25** |
|  |  |  | Middle occipital gyrus | L | -33 -90 20 | 2.76* |
|  |  | 6 | Lingual gyrus (BA18: 90%) | R | 15 -80 -8 | 2.89* |
|  |  |  | Lingual gyrus  (hOC3(V3v): 80%) | R | 21 -80 -12 | 2.59* |
|  | Occipital/  Parietal lobe | 27 | Middle occipital gyrus (IPC(PGp): 40%) | R | 47 -77 11 | 3.09** |
|  | Parietal lobe | 6 | Superior parietal lobule (SPL(7p)=70%) | R | 21 -70 59 | 2.96* |
|  |  | 7 | Precuneus (SPL(7a): 30%) | R | 8 -61 46 | 2.87* |
|  |  | 6 | Superior parietal lobule (SPL(7a):90%) | L | -33 -67 59 | 2.78* |

Notes:
T-values of maxima from the comparisons are given. x, y, z: MNI-coordinates in mm;
L, R left and right hemisphere.
Threshold: p< .01 (uncorr.); spatial extend: 5 voxel;
** p ≤ .001 (uncorr.); * p ≤ .01 (uncorr.)

Cytoarchitectonic probabilities are given according the Anatomy Toolbox 1.8 implemented in SPM8 .

Abbreviations:
BA: Brodman Area; hOC3c(V3v): human occipital cortex. V3 ventral part ; IPC(PGp: Inferior parietal cortex area PGp ; SPL(7P, 7a): superior parietal lobule area 7a, 7p .

Table S4: Brain areas and stereotaxic coordinates for the subliminally presented images of women

| **Com-parison** | **Brain area** | **Cluster**  **Size**  **[voxel]** | **Anatomical region**  Cytoarchitectonic probability | **R/L** | **x y z** | **Significance**  **T** |
| --- | --- | --- | --- | --- | --- | --- |
| **Pretest > Posttest** | Parietal lobe | 1763 | Inferior parietal cortex=IPC(PF): 40% | L | -59 -26 49 | 5.40** |
|  |  |  | Middle cingulate cortex (BA6:40%) | L | -8 -6 46 | 4.64** |
|  |  |  | Supramarginal gyrus (IPC(PF): 70% | L | -62 -29 33 | 4.47** |
|  |  |  | Postcentral gryus (BA4a): 60% | L | -40 -29 65 | 4.20** |
|  |  |  | Superior parietal lobule (SPL(7P):30% | L | -17 -74 49 | 4.09** |
|  |  |  | Precuneus | L | -1 -74 56 | 4.07** |
|  |  |  | Middle temporal gyrus | L | -43 -51 20 | 3.93** |
|  |  |  | Middle cingulate cortex (BA6: 30%) | R | 1 -6 43 | 3.91* |
|  |  | 117 | Inferior parietal lobule (IPC(PFt): 40% | R | 56 -29 52 | 3.56** |
|  |  |  | Supramarginal gyrus (IPC(PFm: 60%) | R | 60 -42 30 | 3.49** |
|  |  |  | Postcentral gyrus (BA1: 90%) | R | 50 -32 59 | 3.40** |
|  |  |  | Angular gyrus (IPC(PGa) 90%) | R | 63 -54 24 | 3.24** |
|  |  | 6 | Rolandic operculum (OP4: 40% | L | -52 -3 4 | 3.09** |
|  |  | 6 | Superior parietal lobule (SPL(5L): 40% | R | 18 -54 65 | 2.82* |
|  | Temporal lobe | 23 | Superior temporal gyrus (IPC(PF): 50% | L | -62 -32 20 | 4.36** |
|  |  |  | Postcentral gyrus (OP1: 40%) | L | -61 -16 14 | 2.52* |
|  |  | 59 | Middle temporal gyrus | R | 56 -48 -5 | 4.09** |
|  |  | 32 | Temporal pole | L | -49 13 -2 | 3.76** |
|  |  | 8 | Fusiform gyrus | L | -43 -51 -24 | 3.52** |
|  |  | 29 | Middle temporal gyrus | R | 50 -48 11 | 3.01* |
|  |  | 25 | Middle temporal gyrus | L | -46 -61 1 | 3.06** |
|  | Occipital lobe/  Cerebellum | 391 | Calcarine gyrus (BA17: 60%) | R | 24 -67 8 | 4.09** |
|  |  |  | Cerebellum | R | 5 -67 -24 | 3.93** |
|  |  |  | Calcarine gyrus (BA 17: 70%) | L | -4 -83 11 | 3.66** |
|  |  |  | Cerebellum | L | -8 -64 -8 | 3.63** |
|  | Occipital lobe | 12 | Middle occipital gyrus | L | -43 -80 8 | 3.22** |
|  | Orbito-frontal lobe | 31 | Middle orbital gyrus | R | 37 61 -8 | 3.89** |
|  |  |  | Superior orbital gyrus | R | 31 61 -8 | 3.86** |
|  |  |  | Middle frontal gyrus | R | 37 64 1 | 3.29** |
|  |  | 10 | Middle orbital gyrus | L | -40 58 -8 | 3.44** |
|  |  | 62 | Rectal gyrus | R | 8 32 -18 | 3.19** |
|  |  |  | Rectal gyrus | L | 2 26 -18 | 3.07** |
|  |  |  | Superior orbital gyrus | L | -14 38 -15 | 2.79* |
|  |  |  | Medial orbital gyrus | L | -8 32 -12 | 2.51* |
|  | Frontal lobe | 394 | Middle frontal gyrus | R | 37 6 46 | 3.83** |
|  |  |  | Inferior frontal gyrus (BA44: 50%) | R | 47 10 24 | 3.78** |
|  |  |  | Precentral gyrus | R | 37 0 36 | 3.72** |
|  |  |  | Inferior frontal gyrus (BA45: 40% | R | 47 22 20 | 3.50** |
|  |  |  | Supplementary area (BA6: 30%) | R | 15 6 62 | 2.80* |
|  |  | 32 | Inferior frontal gyrus (BA44. 45: 20%) | L | -43 22 27 | 3.77** |
|  |  | 82 | Middle frontal gyrus | R | 34 45 30 | 3.12** |
|  |  | 11 | Rolandic operculum (BA44: 40%) | R | 60 6 4 | 2.82* |
|  |  | 36  12 | Middle frontal gyrus | L | -33 51 24 | 2.79* |
|  |  |  | Superior medial gyrus | R | 8 45 33 | 2.68* |
|  | Thalamus | 33 | Thalamus (Th-parietal: 91%) | L | -20 -26 4 | 2.80* |
|  |  | 9 | Thalamus (Th-prefrontal: 90%) | R | 12 -10 8 | 2.82* |
|  | Insula | 24 | Insula lobe | R | 31 22 8 | 3.15** |
|  |  | 5 | Insula lobe | L | -30 13 11 | 3.07** |
|  | Cingulate cortex | 25 | Middle cingulate cortex | R | 12 26 30 | 2.95* |
| **Posttest > Pretest** | Occipital lobe | 138 | Cuneus (BA18: 20%) | R | 12 -90 33 | 4.30** |
|  |  |  | Superior occipital gyrus (BA18: 20%) | R | 15 -93 30 | 4.27** |
|  |  |  | Calcarine gyrus (BA17: 100%) | R | 8 -90 1 | 3.15** |
|  |  |  | Calcarine gyrus (BA17: 90%) | L | 5 -86 -5 | 3.11** |
|  |  | 45 | Superior occipital gyrus | L | -14 -90 20 | 3.77** |
|  |  |  | Middle occipital gyrus (BA18: 20%) | L | -20 -96 17 | 3.69** |
|  | Temporal | 62 | Fusiform gyrus (hOC4(V4): 80% | R | 28 -74 -15 | 3.98** |
|  | Orbito-frontal lobe | 8 | Medial orbital gyrus | R | 5 51 -8 | 2.70* |
|  |  |  |  |  |  |  |

Notes:
T-values of maxima from the comparisons are given. x, y, z: MNI-coordinates in mm;
L, R left and right hemisphere.
Threshold: p < .01 (uncorr.), spatial extend: 5 voxel;
**p ≤ .001(uncorr.); * p ≤ .01 (uncorr.)
Cytoarchitectonic probabilities are given according the Anatomy Toolbox 1.8 implemented in SPM8 .

Abbreviations:
BA: Brodman Area; IPC(PGa, PGp, PF, PFt, PFm,): Inferior parietal cortex area PGa, PGp, PF, PFt or PFm ; hOC3c(V3v): Human occipital cortex V3 ventral part ; hOC4(V4): Human occipital cortex V4 ; OP: Operculum; SPL(7P,7a): Superior parietal lobule area 7a, 7p

References

1. Stoléru S, Fonteille V, Cornélis C, Joyal C, Moulier V: **Functional neuroimaging studies of sexual arousal and orgasm in healthy men and women: A review and meta-analysis.** *Neuroscience and Biobehavioral Reviews* 2012, **36:**1481-1509.

2. Jordan K, Fromberger P, Muller JL: **Neurobiologie der Sexualität und sexueller Störungen.** In *Praxisbuch Sexuelle Störungen.* Edited by Briken P, Berner Michael M. Stuttgart: Thieme Verlag; 2013

3. Ellis SR, Smith JD: **Patterns of statistical dependency in visual scanning.** In *Eye movements and human information processing.* Edited by Groner R, McConkie GW, Menz C. Amsterdam: Elsevier; 1985: 221-238

4. Caseras X, Garner M, Bradley BP, Mogg K: **Biases in visual orienting to negative and positive scenes in dysphoria: An eye movement study.** *Journal of Abnormal Psychology* 2007, **116:**491-497.

5. Gao X, Wang Q, Jackson T, Zhao G, Liang Y, Chen H: **Biases in orienting and maintenance of attention among weight dissatisfied women: an eye-movement study.** *Behavior Research and Therapy* 2011, **49:**252-259.

6. Fromberger P, Jordan K, von Herder J, Steinkrauss H, Nemetschek R, Stolpmann G, Müller JL: **Initial orienting towards sexually relevant stimuli: Preliminary evidence from eye movement measures.** *Archives of Sexual Behavior* 2012, **41:**919-928.

7. Fromberger P, Jordan K, Steinkrauss H, von Herder J, Witzel J, Stolpmann G, Kröner-Herwig B, Müller JL: **Diagnostic accuracy of eye movements in assessing pedophilia.** *The Journal of Sexual Medicine* 2012, **9:**1868-1882.

8. Lang PJ, Bradley MB, Cuthbert BN: **International Affective Picture System, 2001.** NIMH Center for the Study of Emotion and Attention: University of Florida, Gainesville, FL 2001.

9. Eickhoff SB, Stephan KE, Mohlberg H, Grefkes C, Fink GR, Amunts K, Zilles K: **A new SPM toolbox for combining probabilistic cytoarchitectonic maps and functional imaging data.** *Neuroimage* 2005, **25:**1325-1335.

10. Eickhoff SB, Heim S, Zilles K, Amunts K: **Testing anatomically specified hypotheses in functional imaging using cytoarchitectonic maps.** *NeuroImage* 2006, **32:**570-582.
